# Protocol for a home-based self-delivered prehabilitation intervention to proactively reduce fall risk in older adults: a pilot randomized controlled trial of transcranial direct current stimulation and motor imagery

**DOI:** 10.1186/s40814-024-01516-1

**Published:** 2024-06-14

**Authors:** Clayton W. Swanson, Sarah E. Vial, Todd M. Manini, Kimberly T. Sibille, David J. Clark

**Affiliations:** 1 Department of Neurology, College of Medicine, University of Florida, Gainesville, FL USA; 2 Brain Rehabilitation Research Center, Malcom Randall VA Medical Center, Gainesville, FL 32603 USA; 3 Department of Health Outcomes & Biomedical Informatics, College of Medicine, University of Florida, Gainesville, FL USA; 4 Department of Physical Medicine and Rehabilitation, College of Medicine, University of Florida, Gainesville, FL USA

**Keywords:** Aging, Falls, Motor imagery, tDCS, Mobility, Feasibility, Acceptability

## Abstract

**Background:**

Several changes occur in the central nervous system with increasing age that contribute toward declines in mobility. Neurorehabilitation has proven effective in improving motor function though achieving sustained behavioral and neuroplastic adaptations is more challenging. While effective, rehabilitation usually follows adverse health outcomes, such as injurious falls. This reactive intervention approach may be less beneficial than prevention interventions. Therefore, we propose the development of a prehabilitation intervention approach to address mobility problems before they lead to adverse health outcomes. This protocol article describes a pilot study to examine the feasibility and acceptability of a home-based, self-delivered prehabilitation intervention that combines motor imagery (mentally rehearsing motor actions without physical movement) and neuromodulation (transcranial direct current stimulation, tDCS; to the frontal lobes). A secondary objective is to examine preliminary evidence of improved mobility following the intervention.

**Methods:**

This pilot study has a double-blind randomized controlled design. Thirty-four participants aged 70–95 who self-report having experienced a fall within the prior 12 months or have a fear of falling will be recruited. Participants will be randomly assigned to either an active or sham tDCS group for the combined tDCS and motor imagery intervention. The intervention will include six 40-min sessions delivered every other day. Participants will simultaneously practice the motor imagery tasks while receiving tDCS. Those individuals assigned to the active group will receive 20 min of 2.0-mA direct current to frontal lobes, while those in the sham group will receive 30 s of stimulation to the frontal lobes. The motor imagery practice includes six instructional videos presenting different mobility tasks related to activities of daily living. Prior to and following the intervention, participants will undergo laboratory-based mobility and cognitive assessments, questionnaires, and free-living activity monitoring.

**Discussion:**

Previous studies report that home-based, self-delivered tDCS is safe and feasible for various populations, including neurotypical older adults. Additionally, research indicates that motor imagery practice can augment motor learning and performance. By assessing the feasibility (specifically, screening rate (per month), recruitment rate (per month), randomization (screen eligible who enroll), retention rate, and compliance (percent of completed intervention sessions)) and acceptability of the home-based motor imagery and tDCS intervention, this study aims to provide preliminary data for planning larger studies.

**Trial registration:**

This study is registered on ClinicalTrials.gov (NCT05583578). Registered October 13, 2022. https://www.clinicaltrials.gov/study/NCT05583578

## Background

The effects of aging on the central nervous system are multifaceted and result in numerous impairments that impact mobility performance, cognitive function, quality of life, and independence. A consequence of these age-related impairments is the heightened risk of falls and fall-related injuries, which are common among adults 65 years of age and older [1]. Thirty percent of older adults experience a fall annually, with 20–30% of these falls causing moderate-to-severe injuries [2, 3]. In the United States, falls result in over 3 million emergency room visits, 800,000 hospitalizations, and 30,000 deaths annually, amounting to roughly 8% of the annual Medicaid budget being spent on fall-related injury treatments [1, 4, 5]. While falls requiring medical attention are well documented, there are an even larger number of falls that go unreported. Despite the attention directed toward fall prevention, they continue to pose substantial risk among adults living with and without assistance, particularly in those older adults trying to maintain independence.

The challenge in mitigating fall risk is not a lack of effective interventions, but rather that most interventions are reactive to a fall that has already occurred. As such, many interventions are designed for older adults who are characterized as having a moderate fall risk. Furthermore, such interventions are often intensive, requiring clinical expertise and one-on-one care focused on improving exercise capacity and motor coordination. Even with the most established rehabilitation methods, producing durable improvements for older adults remains a challenge. As such, developing a proactive fall mitigation approach requiring less burden on clinicians and older individuals could prove beneficial. This is especially true when incorporating resource-intensive neurorehabilitation methods that promote and enhance motor learning and neuroplasticity but traditionally require considerable therapist involvement.

One method of promoting motor learning that requires less therapist involvement is the use of motor imagery (MI) which refers to the combined practice of action observation and mental imagery. Action observation and mental imagery are frequently implemented to promote and enhance motor learning and are particularly effective when practiced together in series [6]. Action observation refers to the act of watching another individual perform a task. This allows the observer to obtain information about that task’s requirements by watching and evaluating the motor strategies necessary for completion [7–9]. Mental imagery refers to the act of mentally rehearsing a motor task and the accompanied sensations of that task, without physically moving [10–12]. Studies have shown that both action observation and mental imagery augment motor performance for upper limb, lower limb, and whole-body functional tasks [12–16]. Furthermore, research indicates that MI in motor behavior and motor learning paradigms facilitate activity-dependent neuroplastic adaptation which is fundamental to achieving lasting behavioral change [17–19]. For instance, functional brain imaging studies have demonstrated overlapping neural networks (e.g., frontoparietal network) and similar neural connectivity patterns in primary motor and motor-associated brain regions when comparing action observation and mental imagery to physical practice [19–22]. Additionally, corticospinal excitability has shown comparable changes in response to MI or physical practice of complex motor tasks [23–25]. Moreover, low levels of neuromuscular activation have been observed in the absence of movement during imagined arm flexion (i.e., dumbbell curling), exhibiting similar muscle activation patterns (although with much lower activation amplitudes) for nine upper limb muscles fundamental to the task (i.e., agonist, antagonist, synergist, and fixator muscles) [26]. Furthermore, differential neuromuscular responses were recorded for imagined “heavy” vs. “light” dumbbell lifts [26]. Together, these studies indicate that MI engages similar neural circuits and neuromuscular activation patterns as physical performance.

Transcranial direct current stimulation (tDCS) is a noninvasive brain stimulation technique which induces a relatively weak electrical current to a targeted region of the brain, influencing neuronal membrane potentials [27, 28]. This method of neuromodulation shows promising results for modifying cortical excitability and facilitating motor learning [29]. For instance, after a single session, participants receiving active tDCS during a motor learning paradigm demonstrated greater motor improvements compared to participants receiving sham stimulation. Multiple stimulation sessions facilitated superior skill retention resulting in sustained improvements up to 3 months post-intervention [30]. Moreover, concurrent task practice and stimulation appear to induce the greatest motor improvements [13, 31]. For example, Saruco and colleagues have shown that excitatory tDCS during a postural stability (i.e., balance) MI paradigm significantly improved postural stability performance when compared to the sham tDCS group [31]. Indicating that MI practice combined with tDCS may produce a robust activation of neural circuits important to task performance, thus driving a greater neuroplastic response.

Due to technological advancements over that past decade, the capacity to monitor and intervene within the home has drastically improved. These advances provide opportunities for developing new approaches that target mobility and independence for older adults. Studies have demonstrated that home-based, self-delivered tDCS is safe and feasible for various populations including neurotypical older adults [32, 33]. Although MI may not be superior to conventional physical practice or rehabilitation, it may provide an effective, convenient, and safe alternative for practicing complex walking conditions within the confines of one’s own home. Therefore, our study aims to assess feasibility and acceptability of a home-based, self-delivered MI and tDCS intervention for improving mobility in fall-prone older adults. Additionally, we will collect preliminary data to investigate the effect of MI and active tDCS on mobility function.

## Methods/design

### Study design

**Fig. 1:**
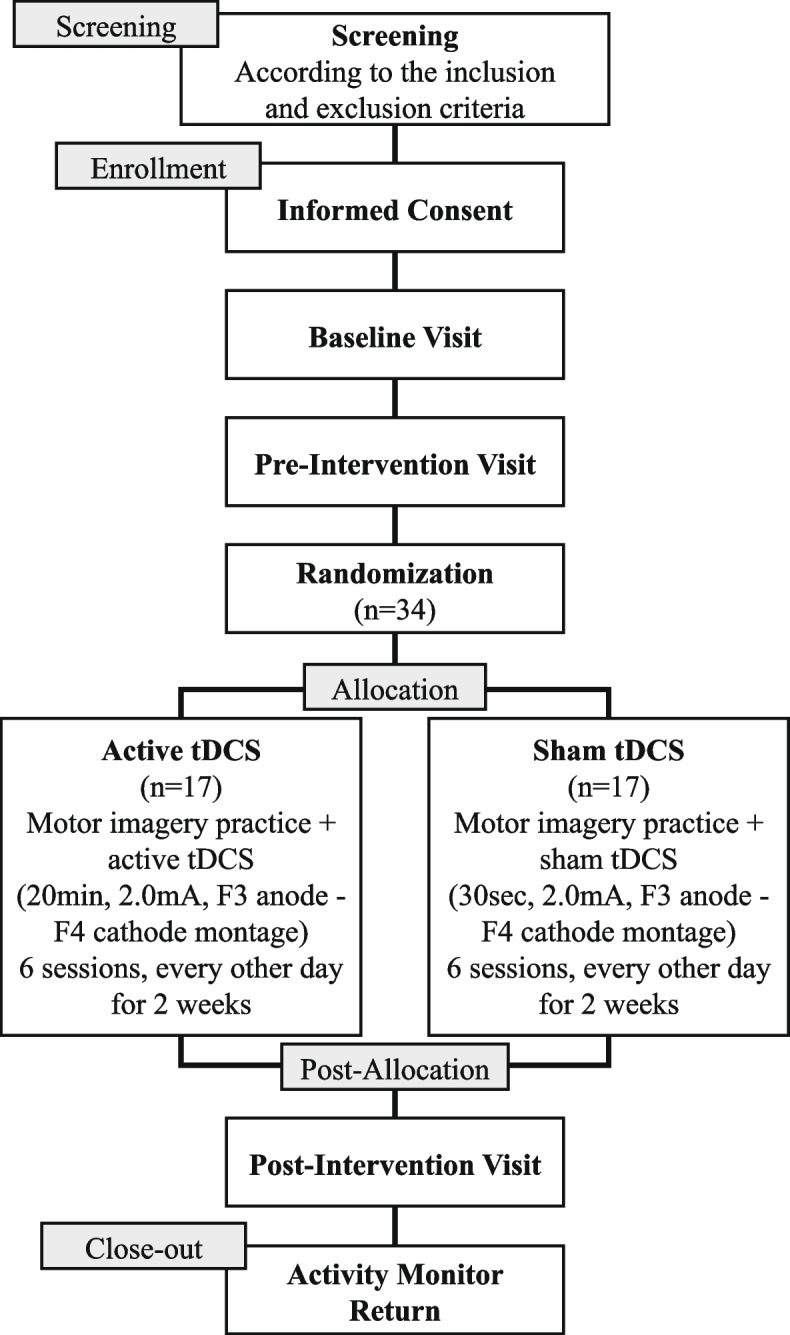
Study flowchart

This study is a double-blind, randomized controlled pilot trial designed according to the Consolidated Standards of Reporting Trials (CONSORT) extension to pilot trials [34]. The study flowchart is shown in Fig. 1. Thirty-four adults aged 70– 5 will be enrolled and randomly allocated to an active or sham tDCS group. This study protocol was approved by the University of Florida (UF) Institutional Review Board (Study ID: IRB202201802) and was registered on ClinicalTrials.gov (NCT05583578). This trial is funded by the UF Claude D. Pepper Older Americans Independence Center (NIH/NIA: P30AG028740).

### Participant selection

Participants will be recruited via mailers sent to participants in the UF Pepper Center Registry, posted recruitment flyers, and word of mouth. Individuals interested in the study will complete a telephone screening. Initial eligibility includes participants aged 70–95 having a self-reported fall risk. Criteria for fall risk is determined by whether the individual had (and recovered from) a fall-related injury in the previous year, had fallen two or more times in the previous year, or reports a fear of falling due to balance and walking limitations [35]. Additionally, individuals will be asked if they had experienced any trips or stumbles over the last year requiring a corrective response (e.g., grabbing the wall as to not hit the ground). The enrollment criteria were designed to be inclusive to ensure generalizability of the results, although participants were required to be medically stable and free of diagnosed neurological injury or disease. Eligibility is determined by the following criteria: (1) willing to be randomized into either study group, (2) living in the community and able to travel to research site, (3) able to independently assemble and place the tDCS headband or incorporate the involvement of a willing study partner, (4) willing and able to provide informed consent. Exclusion criteria include the following: (1) a diagnosed neurological disorder, injury of the central nervous system, or observed symptoms consistent with such a condition (i.e., spinal cord injury, Alzheimer’s, Parkinson’s); (2) a score of 23 or less on the Montreal Cognitive Assessment (MoCA); (3) contraindications to noninvasive brain stimulation (e.g., metal in the head); (4) medications affecting the central nervous system including, but not limited to, benzodiazepines, anticholinergic medications, and GABAergic medications; (5) severe arthritis, such as awaiting joint replacement; (6) uncontrolled or controlled cardiovascular disease that limits the participants ability to complete light aerobic mobility assessments; (7) lung disease requiring supplemental oxygen; (8) renal disease requiring dialysis; (9) uncontrolled diabetes; (10) terminal illness; (11) myocardial infarction or major heart surgery in the previous year; (12) cancer treatment in the past year, except for nonmelanoma skin cancers and cancers having an excellent prognosis; (13) current diagnosis of a psychotic disorder, schizophrenia, or bipolar disorder; (14) unable to communicate with study personnel; (15) uncontrolled hypertension at rest (systolic > 180 mmHg and/or diastolic > 100 mmHg); (16) bone fracture or joint replacement in the previous six months; (17) current participation in physical therapy or cardiopulmonary rehabilitation; (18) current enrollment in a clinical trial that may influence the results of either study; and (19) clinical judgment of investigative team.

### Group randomization

Group allocation will be randomized and counterbalanced ensuring similar stimulation group allocation numbers throughout enrollment. The randomization assignment will be predetermined through a computer program which an unblinded study coordinator will manage. The unblinded coordinator will oversee programing the tDCS units for active or sham stimulation and documenting the stimulator activation codes which will then be provided to the study staff and participants.

### Sample size

This study is designed to assess feasibility and acceptability of a home-based, self-delivered motor imagery and tDCS intervention and to acquire preliminary data to plan and conduct power analyses for a larger subsequent study. Following Whitehead and colleagues “stepped rule of thumb” recommendations for determining pilot sample size and because actual effect sizes were unknown while planning this study, we anticipated achieving a medium effect size range (i.e., Cohen’s *d* = 0.3–0.7) for mobility-related secondary outcome measures (see Table 1) [36]. Therefore, a total sample of 34 participants would provide enough data to estimate a main trial sample size with 90% power granted the pilot study produces effect sizes that fall within the anticipated range [36]. The proposed pilot sample may be adjusted to include additional participants based on recruitment interest, capacity, and time.

**Table 1:** Study outcomes

Primary outcome measures
*• Acceptability* *Intervention device interaction rating* — Likert scale (from 0 [strongly disagree] to 10 [strongly agree]) composed of nine questions regarding participants use of home-based tDCS and MI. For example, question 1 “It was easy to prepare the device and accessories for each session,” 2 “The device and setup was unnecessarily complex,” 4 “I felt the videos and movements covered on the videos were helpful,” 7 “I felt confident using the device,” and 8 “I needed to learn a lot of things before I could get going with this device.” Acceptability will be evaluated across groups by reporting descriptive measures of satisfaction ratings [37]. *tDCS stimulation rating* — Likert scale (from 0 [nothing/none] to 10 [strongest/worst possible]) composed of commonly experienced tDCS side effects (e.g., tingling, itching, burning, pain, fatigue, nervousness). tDCS sensation acceptability across groups will be evaluated by reporting descriptive measures of tDCS side effects.
*• Feasibility* Screening rate (per month), recruitment rate (per month), randomization (screen eligible who enroll), retention rate, and compliance (percent of completed intervention sessions) [38]
Secondary outcome measures
*• Questionnaires* ABC scale [39], MSRS [40], PSQI [41], MIQ-RS [42], SF-36 [43], Katz ADL [44], CHAMPS [45], Intervention-Specific Motor Imagery Scale
*• Neuropsychological tests* Computer Based Neurocognitive Assessment [46], MoCA [47], TMT (a & b) [48]
*• Functional mobility* mBEST [49], CTSIB [50], 2MTW (self-selected natural pace, self-selected natural pace dual-task, self-selected fast pace), 360° turn test
*• Physical activity* Total steps, total sedentary time, and physical activity score (MET.h)

Abbreviations: *ABC* scale, Activities Specific Balance Confidence Scale; *MSRS*, Movement-specific Reinvestment Scale; *TMT*, Trail Making Test; *PSQI*, Pittsburgh Sleep Quality Index; *MIQ-RS*, Motor Imagery Questionnaire (revised second edition); *SF-36*, short form-36; Katz ADL, Katz Index of Independence; *CHAMPS*, Community Health Model Activities Program for Seniors Physical Activity Questionnaire; *MoCA*, Montreal Cognitive Assessment; *mBEST*, Mini Balance Evaluation Systems Test; *CTSIB*, Clinical Test of Sensory Interaction on Balance; *2MWT*, 2-min walk test; *MET*.h, metabolic equivalents

### Experimental protocol

**Fig. 2:**
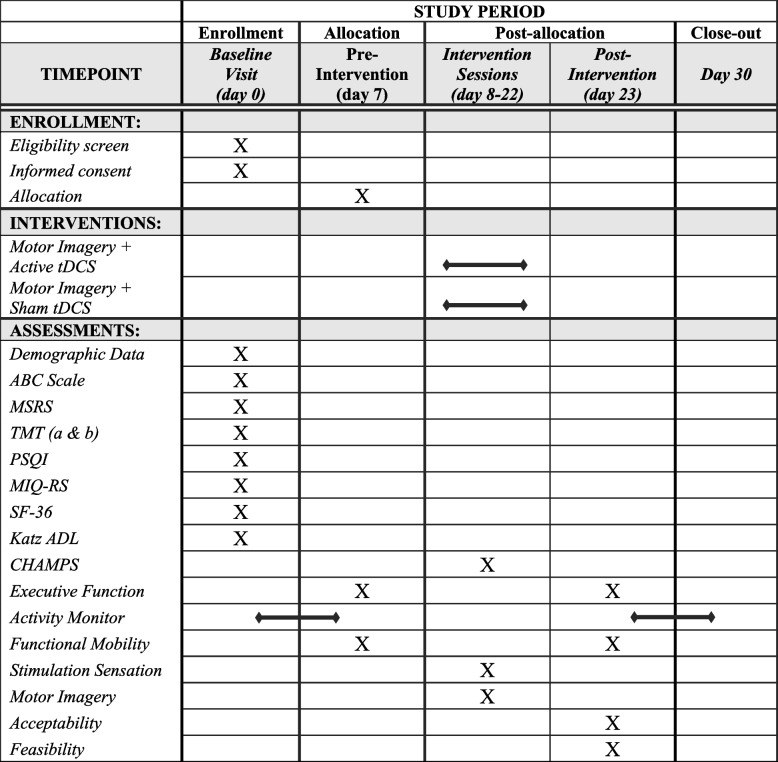
SPIRIT figure. Participant schedule of enrollment and data collection. ABC scale, Activities specific Balance Confidence Scale; MSRS, Movement-specific Reinvestment Scale; TMT, Trail Making Test; PSQI, Pittsburgh Sleep Quality Index; MIQ-RS, Motor Imagery Questionnaire (revised second edition); SF-36, short form-36; Katz ADL, Katz Index of Independence; CHAMPS, Community Health Model Activities Program for Seniors Physical Activity Questionnaire

The data collection timeline is summarized in the Standard Protocol Items: Recommendations for Interventional Trials (SPIRIT) figure (Fig. 2). Following a telephone screen, those who qualify and complete the informed consent form will be assessed in the laboratory at three distinct time points (baseline, pre-intervention, and post-intervention) along with completing the home-based MI and tDCS intervention.

### Baseline visit

At the beginning of the baseline visit, study personnel and individuals will complete the informed consent form. Once signed, participants will complete a series of questionnaires including the Activities Specific Balance Confidence Scale, the Movement-specific Reinvestment Scale, Trail Making A & B, the Pittsburgh Sleep Quality Index, the Motor Imagery Questionnaire (revised second edition), the Short Form-36, and the Katz Index of Independence which will be used as secondary outcome measures. Following the questionnaires, participants will leave wearing the validated ActivPAL activity monitor (ActivPAL4, PAL Technologies Ltd., UK) [51, 52]. To ensure freedom of movement while at home, these lightweight, small, and unobtrusive triaxial accelerometers will be placed in a waterproof sleeve and secured to the thigh using a Tegaderm^™^ adhesive dressing (3M, London, ON, Canada). As secondary movement outcomes, we plan to assess total steps, total sedentary time, and participants physical activity score measured in metabolic equivalents (MET.h). After wearing the activity monitor for 7 to 9 days, participants will return for the pre-intervention visit.

### Pre-intervention visit

At the beginning of the pre-intervention visit, the activity monitor will be removed from the participants thigh. Participants will then complete a series of computer-based neurocognitive tests to assess multiple aspects of executive function including verbal short-term memory, response inhibition, attention, visuospatial working memory, visuospatial processing, and spatial short-term memory [46].

Upon completion of the neurocognitive tests, participants will perform several clinically feasible mobility assessments showing strong psychometric properties [53–58]. Specifically, participants will complete the Mini-Balance Evaluation Systems Test (mBEST) which is a 14-item mobility performance examination categorized to assess static and dynamic balance by assessing four sub-domains of mobility (i.e., anticipatory, reactive postural control, sensory orientation, and dynamic gait) [49, 59]. In addition, participants will complete the Clinical Test of Sensory Interaction on Balance (CTSIB) which involves four 30-s trials each intended to assess unique components of balance. To assess walking, participants will perform three individual two-minute walk test (2MWT) trials, including a self-selected natural pace, a self-selected fast pace, and a self-selected natural pace dual task (serial sevens) walk. Lastly, participants will perform a series of three 360° turns at their self-selected natural pace and self-selected fast pace.

The mBEST will be scored following the published scoring guidelines which allows for a maximum score of 28 from the 14 tasks. Each task is scored on a ordinal scale ranging between 0 (indicating the lowest level of function) and 2 (indication the highest level of function) [59]. In addition, and simultaneously, all mobility assessments will be instrumented via wireless inertial sensors (APDM Inc., Portland, OR, USA) securely placed on each foot, around the waist (lumbar level L4-L5), on the sternum, and on the forehead. Between each mobility test, data from the sensors will be wirelessly streamed to a laboratory computer and processed using validated software (Mobility Lab v2, Portland, OR, USA), thus providing a variety of spatiotemporal kinematic outcomes for assessing movement quality [60]. Both the subjective mBEST score and the objective mobility measures will be used as secondary outcome measures to characterize mobility. Lastly, participants will undergo a detailed familiarization training with research staff to help ensure proper implementation of the home-based intervention protocol. During the training, participants will become familiar with all aspects of the tDCS setup (i.e., securing sponge electrodes, placing head strap correctly and accurately, and initializing the stimulator), accessing the motor imagery videos, and completing the intervention specific questionnaires (i.e., the simulation sensation, motor imagery, and the Community Health Model Activities Program for Seniors Physical Activity (CHAMPS) Questionnaire).

### Intervention sessions

Following the pre-intervention visit, participants will complete the MI and tDCS intervention. The home-based, self-delivered intervention will consist of six 40-min sessions over the course of 2weeks (one session every other day).

#### Motor imagery

**Fig. 3:**
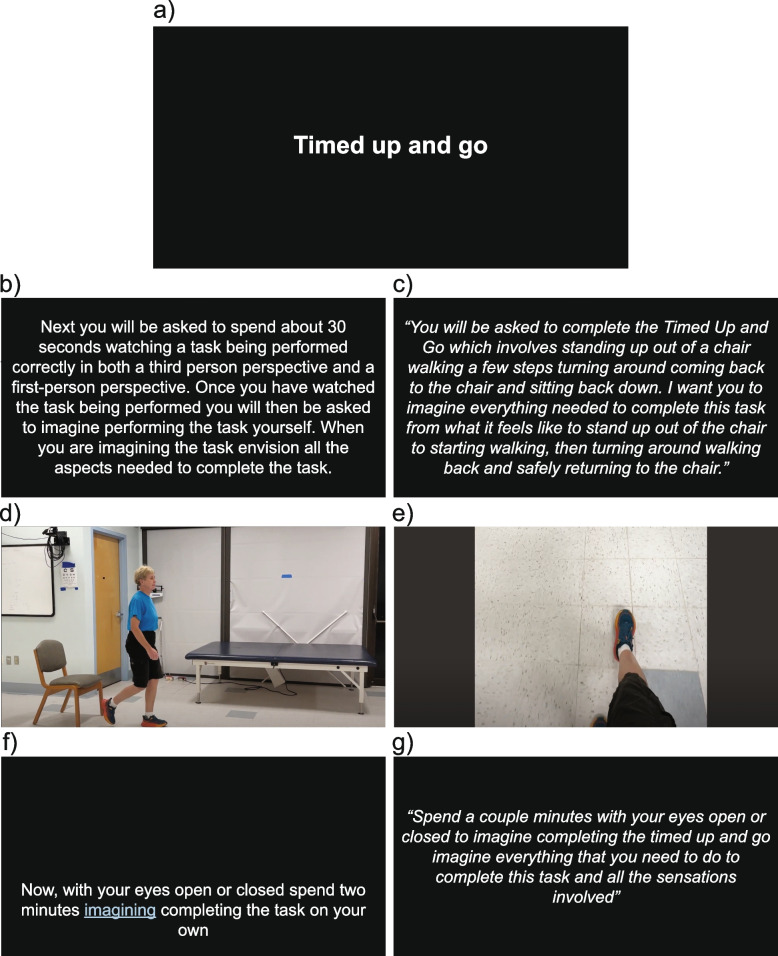
Examples from the **a** Timed Up and Go mental imagery practice video. **b** Written instruction about what participants should expect next. **c** Verbal instruction specific to the task participants are about watch. **d** Third person perspective of the task being performed correctly. **e** First-person perspective of the task being performed correctly. **f** Written instruction for the imagined practice. **g** Verbal instruction for the imagined practicing

Each of the six intervention sessions will include MI practice. The tasks that participants will practice are from clinically based functional mobility assessments, which demonstrate strong psychometric properties and resemble activities of daily living (e.g., sit to stand, 360° turn, walking and turning, and balancing) [54–58]. We will also include practice of an ecologically applicable outdoor walking task involving changing terrains (e.g., transitioning from grass to a concrete sidewalk). Participants will be provided a YouTube hyperlink, providing them access to the different videos. During the first portion of each video, participants will receive written and verbal guidance on what aspects of each movement to watch and mentally rehearse (Fig. 3b and c). Then each video will portray a specific task being performed correctly from both a third-person (Fig. 3d) and first-person (Fig. 3e) perspective. After watching the task being performed correctly, participants will be instructed to rehearse the task mentally without any corresponding physical movement (Fig. 3f and g). This sequence of action observation followed by mental imagery facilitates the vividness of the motor imagery practice. Participants will spend 4 to 5 min mentally practicing each task for a total of 40 min per session.

#### Transcranial direct current stimulation (tDCS)

Participants will be randomized and counterbalanced (i.e., equal numbers in each group) to receive either active or sham tDCS, which will be delivered simultaneously during the MI practice. A Soterix 1 × 1 mini-clinical trials tDCS unit will be used for delivery of stimulation (Soterix Medical Inc.). Participants will be provided single-use Soterix “SNAPpad” sponge electrodes (5 × 7 cm) which are individually sealed and pre-saturated with a conductive saline solution and snap onto the pre-configured Soterix head strap. To ensure consistent sponge electrode placement, participants will be provided a measuring tool specifically configured to their head along with hands-on practice and instructional documentation (i.e., picture diagrams and videos). This will help ensure participants are familiar with snapping the electrodes and placing the head band at home. To further ensure participants can place the headband and initialize each tDCS session, the study staff will coordinate with the participant to guarantee a staff member is available by telephone to answer any questions.

Participants will also be provided instructions for operating the handheld tDCS device. Each participant will be provided six unique single-use 5-digit activation codes which will be used to initiate the stimulation for each intervention session. Only the unblinded study coordinator will program the device and write down the unique code for each session; therefore, neither the study participant nor the blinded study assessment personnel will know group allocation.

For both active and sham, sponge electrodes will be placed over the frontal cortices at F3 and F4 (based on the international “10–20 system” of standardized brain electrode placement). For the active group, participants will receive 20 min of 2.0-mA direct current stimulation with a 30-s ramp up and ramp down. For the sham stimulation, all procedures will be identical except for the duration of stimulation. Those participants receiving sham stimulation will receive 30 s of 2.0-mA stimulation at the beginning of each intervention session. Since participants habituate to the sensation of tDCS within 30–60 s of stimulation, this procedure provides a similar sensation of active tDCS [61].

### Post-intervention visit

During the post-intervention visit, participants will complete the same computer-based neurocognitive and functional mobility assessments that were performed during the pre-intervention visit. As a primary outcome measure, participants will complete additional questionnaires to assess acceptability of the home-based intervention (Table 1). Similar to other home-based interventions, acceptability will be evaluated through structured questionnaires [37]. Following the intervention, participants will be asked to complete a debriefing questionnaire containing a series of questions aimed at assessing participants satisfaction interacting with the tDCS device and MI videos. Participants will score each question using a Likert scale ranging from 0 (strongly disagree) to 10 (strongly agree). For example, questions will include the following: 1 “It was easy to prepare the device and accessories for each session,” 2 “The device and setup was unnecessarily complex,” 4 “I felt the videos and movements covered on the videos were helpful,” 7 “I felt confident using the device,” and 8 “I needed to learn a lot of things before I could get going with this device.” To gain further information about participants experience in the study, we will ask a series of open-ended questions. These questions include the following: (1) “Are there any movements you wish would have been covered in the MI videos?” (2) “Is there anything that would have made the MI videos more helpful?” and (3) “Is there anything that would have made the tDCS device easier to use?” To assess participants acceptability of tDCS stimulation, they will be asked to complete a stimulation questionnaire before and after each intervention session. This questionnaire will ask participants to rate commonly experienced tDCS side effects (e.g., tingling, itching, burning, pain, fatigue, nervousness) using a Likert scale ranging from 0 (nothing/none) to 10 (strongest/worst possible). At the end of the visit, participants will have the ActivPal monitor secured to their thigh for an additional 7 to 9 days before returning it to the laboratory.

### Feasibility assessment

To assess feasibility and plan for future research studies, we will track screening, recruitment, randomization, retention, and compliance [38]. Screening and recruitment will be quantified as number of people screened and/or enrolled per month. Randomization will be quantified as the proportion of screen eligible participants who enroll. Retention will be quantified by group as the percentage of people who complete the study protocol. Lastly, compliance will be quantified by the percentage of people who complete all intervention sessions. Intervention sessions will be tracked via session questionnaires and confirmed via the tDCS unit’s session log, which participants are not informed of and do not have access to. Feasibility will be considered supported if the trial demonstrates a recruitment rate of 3–4 participants/month using our recruitment strategy and if the retention rate is 80% or higher.

### Analytical methods

For the purposes of this pilot study, the analysis will focus on assessing feasibility and acceptability through descriptive statistics [38, 62]. For categorical variables, statistics will include frequency and percentages, for continuous measures, means, standard deviations, and confidence intervals will be presented. We are not hypothesizing statistical significance (i.e., alpha ≤ 0.05) between groups; rather, we anticipate effect sizes will support a directional effect. For functional mobility and physical activity, we will assess observed effect sizes between active and sham tDCS using Cohen’s d values. Effect sizes will be defined as small (*d* = 0.20), medium (*d* = 0.50), and large (*d* = 0.80) [63].

### Data and safety monitoring

All participants will be de-identified and given a unique identification code that will be associated with their personal information and accessible only to IRB-approved research team members. In terms of tDCS safety, a recent review aggregated results from 488 human-based clinical trials and failed to identify a single record of a serious adverse event related to tDCS in over 1000 subjects receiving > 33,200 sessions over multiple days [64]. Moreover, the review reported that between 10–40% of individuals who received tDCS regardless of group allocation (i.e., active or sham stimulation) experienced effects such as headache, itching, burning sensation (without actual injury), discomfort, and tingling. All these effects dissipate quickly, and tDCS has not been reported to have prolonged negative consequences [64]. While home-based, self-administered tDCS has been shown to be acceptable and safe across various populations, including healthy older adults, participants will be made aware of known potential risks and discomforts during the informed consent process and will be provided time to discuss those risks with a member of the research team. If a participant does not tolerate the stimulation, they will have the opportunity to withdraw from the study. Adverse events will be reported according to the UF IRB guidelines. For tDCS-related adverse events deemed serious and unexpected, unblinding of that participants allocated group may occur if it is relevant to their treatment decisions. For cases where unblinding occurs, the proxy principal investigator (Dr. David Clark) will be unblinded to that participants group allocation. Follow-up considerations will be determined pending IRB review. Adverse events unrelated to tDCS (e.g., a paper cut) will be handled by the principal investigator (Dr. Clayton Swanson). In accordance with UF Research and the Quality Assurance Program, this study may be randomly selected to undergo a quality assurance audit. All members of the research team will undergo training to ensure participant safety and protocol adherence. Members of the research team will meet regularly to monitor and discuss study-related topics.

## Discussion

Visualization techniques such as mental imagery and action observation, especially when performed together during a single session, have demonstrated effectiveness for enhancing motor learning. Functional neuroimaging studies show overlapping regions of cortical activity when comparing MI practice to physical practice, indicating similar neural resources in response to both types of training. Neuromodulatory interventions incorporating similar stimulation parameters to those included in this pilot trial (i.e., 2.0 mA, frontal tDCS montage, multiple sessions) have reported significant effects on motor learning improvements [65]. Our pilot trial aims to assess the feasibility, acceptability, and preliminary efficacy for delivering a home-based, self-delivered MI and tDCS intervention targeting mobility function in fall-prone older adults. The findings of this study will provide the necessary information for planning a larger follow-up trial. Additionally, the information gained will help guide any necessary protocol modifications such as eligibility criteria, intervention dosing, assessment alterations, participant schedules, and sample size predictions.

The present study has some potential limitations. First, the study aims to enroll older adults who have previously experienced a fall or have a recognized fear of falling. As such, determining the correct implementation steps for translating the findings into clinical practice needs to be thoughtfully decided in order to encourage a proactive or prehabilitation emphasis. Second, there are specific activities of daily living that participants are being asked to practice; however, these activities do not capture the full extent of movements made daily. Therefore, it remains unclear whether this study incorporates an adequate sample of movements to successfully train fall prevention. The answer to this question should be addressed in a future study, although data collected from this pilot study may provide initial insights.

This pilot study serves as one of the first home-based, self-delivered MI and tDCS studies targeting mobility function in older adults. We strongly believe that developing a safe, home-based alternative to conventional rehabilitation will provide access to those who may have barriers (e.g., travel restrictions and/or financial limitations) to standard treatments. The results from this pilot study will help inform a larger-scale follow-up study to determine whether this approach can be beneficial for older adults at risk of falling.

## Data Availability

The datasets used and analyzed during the current study are available from the corresponding author upon reasonable request.

## Abbreviations

MI: Motor imagery
tDCS: Transcranial direct current stimulation
MoCA: Montreal Cognitive Assessment
CONSORT: Consolidated Standards of Reporting Trials
UF: University of Florida
SPIRIT: Standard Protocol Items: Recommendations for Interventional Trials
mmHg: Millimeters of mercury
mBEST: Mini-Balance Evaluation Systems Test
CTSIB: Clinical test of sensory interaction on balance
2MWT: Two-minute walk test
MET.h: Metabolic equivalent hours
mA: Milliampere
